# Effect of a Cooperation Strategy between Primary Care Physicians and Hospital Liver Units on HBV Care in Campania, Italy

**DOI:** 10.1155/2018/5670374

**Published:** 2018-07-26

**Authors:** Rosa Zampino, Nicolina Capoluongo, Adriana Boemio, Margherita Macera, Martina Vitrone, Luigi Elio Adinolfi, Pietro Filippini, Evangelista Sagnelli, Caterina Sagnelli, Emanuele Durante-Mangoni, Nicola Coppola

**Affiliations:** ^1^Department of Medical, Surgical, Neurological, Metabolic and Geriatric Sciences, University of Campania “L. Vanvitelli”, Italy; ^2^Unit of Infectious & Transplant Medicine, “V. Monaldi” Hospital, AORN dei Colli, Naples, Italy; ^3^Infectious Diseases Unit, Department of Mental and Physical Health and Preventive Medicine, University of Campania “L. Vanvitelli”, Italy; ^4^Department of Precision Medicine, University of Campania “L. Vanvitelli”, Italy

## Abstract

**Aims:**

This study is aimed at assessing the efficacy of an active search and treat strategy for HBV-infected subjects in an endemic area (Campania, Italy). To do this, we created a cooperation bundle between 24 General Practitioners (GPs) and 3 Hospital Liver Units (HLU). We assessed whether this strategy improved the detection of HBV infection in patients at risk and the overall quality of care, with the aim of reducing liver disease progression.

**Methods:**

We estimated that, among about 20,000 patients cared for by the 24 GPs, approximately 280 patients unaware of or underestimating HBV infection would be found. Identified patients were to be referred to the HLU for clinical evaluation and treatment from February 2016 for 12 months.

**Results:**

Unexpectedly, screening and enrolment were poor (48 patients only). GP workloads, patient financial difficulties, and patients' refusal were the major causes of enrolment failure according to GPs. All patients referred to HLU completed the program; most of them were HBV inactive carriers.

**Conclusions:**

This program failed to scavenge chronic HBV-infected patients in an endemic area and establish a successful clinical collaboration between GPs and HLU. Underlying reasons are diverse and call for new strategies to implement cooperation between primary care providers and hospital specialists.

## 1. Introduction

In recent decades, a progressive reduction in the incidence of acute hepatitis B (AHB) and the prevalence of HBV-related chronic hepatitis has been observed in endemic areas, such as the Campania region of Italy, as a result of improved socioeconomic conditions and universal vaccination [1–3]. The current incidence of AHB is about 1/100,000 inhabitants per year, 15% of cases involving the immigrant population [4], and the prevalence of HBsAg carriers is estimated at nearly 1% in the native population and 3-10% in documented immigrants (regular immigrants who arrived in Italy for economic reasons and have now stable work and residency). It is estimated that 600,000 Italians and nearly 250,000 immigrants are chronic carriers of HBV infection, of whom 50% and 70%, respectively, are unaware of their virological condition [4]. These subjects may transmit HBV infection and remain untreated, even when treatment is warranted [4].

Italian General Practitioners (GPs) are the physicians providing primary care and are usually overwhelmed by a considerable number of patients with symptomatic diseases, leaving them little time for asymptomatic patients, such as most HBsAg-positive patients. Consequently, most of them are not referred to a hepatologist for screening, follow-up, treatment (when necessary), and the implementation of preventive measures [5, 6]. Previous experience showed the benefit of GP involvement in screening strategies to reduce the number of undiagnosed hepatic infections and clarify the prevalence and correlates of the different risk factors [7, 8]. This cooperation could improve the quality of care and the cost-benefit ratio.

We applied a clinical bundle to first actively screen, identify, and treat HBV infection in subjects unaware of their virological condition in Campania, Italy, and refer them to a Hospital Liver Unit (HLU) for clinical evaluation and antiviral treatment, when indicated. A second aim was to create a network between GPs and hepatology specialists to improve the quality of care (diagnosis, clinical evaluation, follow-up, and treatment). The ultimate aim was to reduce the progression of liver disease to cirrhosis, the development of hepatocellular carcinoma (HCC), the risk of in-house and sexual transmission of HBV infection, and the cost of management.

## 2. Methods

We contacted two GP associations in Naples and a group of GPs in Caserta to illustrate the program and seek their participation. One of the GP associations did not join the program because of excessive prior workload, while other GPs gave their full availability to participate. In Caserta, of 15 GPs invited, 5 joined the program. GPs use a database for their clinical records, which we thought would be useful tool to screen data and identify patients' anthropometric measures, clinical history, and current pharmacologic treatment.

Within this network, we tried to apply to a large primary care population methods similar to those successfully used in an ongoing study performed to identify and manage HBV infection in irregular immigrants and refugees [9].

### 2.1. Study Design

The planned duration of the program was one year. On the basis of chronic HBV infection prevalence in Campania [4] and the number of subjects cared for by the GPs participating in this program, we estimated that in a cohort of about 20,000 subjects (at least 20 GPs randomly involved) approximately 280 citizens with HBV infection would be found. An educational meeting with participating GPs was held to discuss the study protocol and review HBV epidemiology, pathophysiology, and treatment. Posters illustrating the “Scavenge HBV in Campania” program were put up in the GPs' offices to inform subjects and sensitize them towards HBV infection. The essential role of the GPs in alerting HBV carriers was stressed. A financial support to participate in the program was given to the GP association.

Each GP was to extrapolate from his/her database subjects cared for with risk factors for HBV infection and/or ALT abnormalities and refer them for screening for serum HBsAg. Each known HBV carrier was to fill out a questionnaire recording age, sex, geographical origin, level of education, religion, family history, cohabitation conditions, sexual habits, history of previous surgery, dental care, tattooing, piercing, drug addiction, blood transfusion, abortion for females, and (when applicable) time of immigration and tribal rituals. GPs had to refer patients with known HBsAg positivity, if not already cared by other HLU.

Family members of subjects found to be HBsAg-positive were to be screened for HBV serum markers. All subjects found to be HBsAg-positive were to be referred at the nearest HLU involved in the program, for clinical examination and the determination of liver function tests, liver ultrasound scan, and HBeAg, anti-HBe, HBV DNA, anti-HDV, anti-HCV, and anti-HIV testing. Fibrosis assessment by noninvasive method (transient elastography, TE; Fibro Scan®, EchoSens, Paris, France) or liver biopsy was to be performed and treatment offered when indicated, in accordance with patient's compliance and current guidelines [10, 11]. Comorbidities and coinfections were to be investigated in depth and treated when indicated.

**Figure 1:**
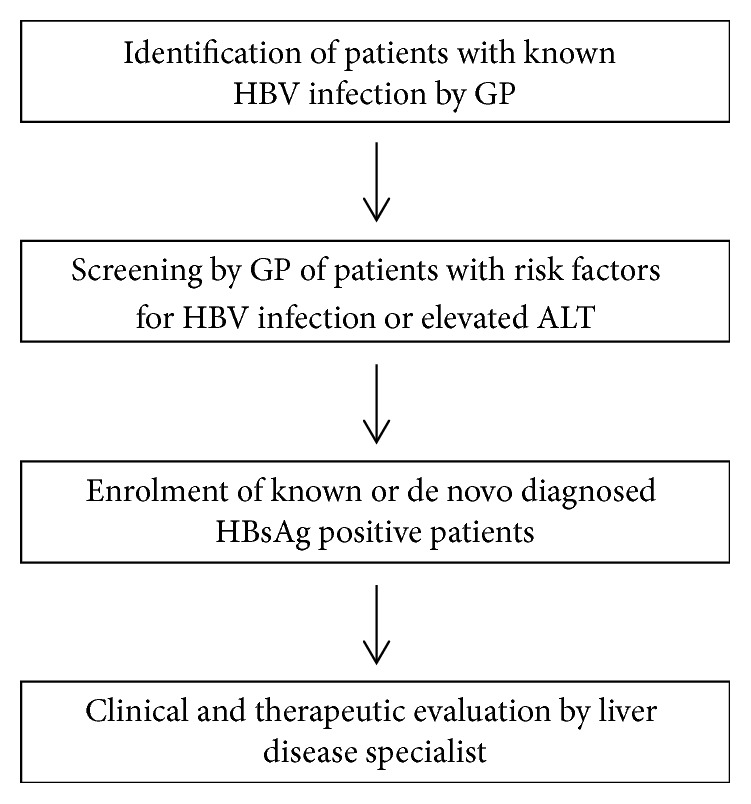
Flowchart of the fundamental phases of the program.

Figure 1 shows the fundamental steps of the program.

All patients, either treated or untreated, were to be followed up with a personalized educational and clinical program. Particular attention was to be given to patients with an advanced liver disease. Cooperation between the GP and the HLU specialist was to be long-lasting to ensure qualified assistance to the HBV subjects and their family members. To favor patient enrolment and adherence, a junior doctor of the investigator's team met with each patient at GP's office and assisted them at their first HLU visit. Moreover, an administrative assistant coordinated visit schedules and kept telephone contacts with doctors and patients to ease required encounters.

Statistical analysis of data was performed using the statistical package STATA to identify risk factors independently associated with HBsAg positivity. The outcome of HBV patients (both treated and untreated) was evaluated according to the criteria suggested in the current AASLD and EASL guidelines [10, 11].

In addition, continued cooperation between GPs and specialists after the project was also assessed.

The study was conducted in accordance with the rules of the Good Clinical Practice and the protocol and procedures were approved by the Ethics Committee of the University of Campania “L. Vanvitelli”.

## 3. Results

**Figure 2:**
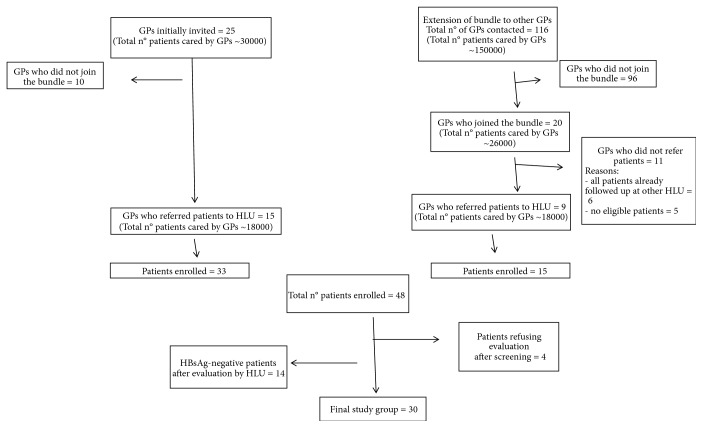
GP involvement in the program.

The program started in February 2016 with the involvement of 25 randomly selected GPs. As only 33 patients from 15 of 25 GP offices were enrolled during the initial 4 months of study, we decided to extend the bundle to all 116 GPs included in the GP association (Figure 2).

### 3.1. Enrolment

The analysis of the GP databases at the beginning of the study identified 282 known HBsAg-positive patients. However, many of these patients either were not involved by GPs or were misclassified as HBsAg. Moreover, less than 50% of patients who were anticipated to have risk factors for HBV infection and/or elevated ALT were screened. The reasons for such a low enrolment rate were reported only by a small number of GPs and are summarized in Table 1. GP workloads (many patients with different symptomatic disorders to follow), patients' financial difficulties, and simple refusal to participate were the most frequently reported. Furthermore, diabetes mellitus and oncohematologic diseases were considered a priority by some GPs compared to possible or asymptomatic liver disease.

Eleven GPs did not refer patients to the specialist liver units stating these were already followed up at other HLU (6 GPs) or due to lack of any HBsAg-positive subject in their patient population (5 GPs) (Figure 2).

An analysis performed by a member of the HLU specialists' team, who was in direct contact with GPs, revealed common mistakes in the GP databases, with patient misclassification regarding ALT values or HBV status or failure to update past test results. In particular, 30% of patients who were meant to be HBsAg-positive were in fact HBsAg-negative. This likely caused the initial overestimation of the number of patients available for the study.

### 3.2. Referral to Hospital Liver Units

Although the percentage of HBsAg-positive subjects referred was much lower than expected, among the identified patients, only 4 declined to continue HLU follow-up after screening.

Overall, 44 referred patients as known or presumed HBsAg-positive started screening at the HLU. Of them, 14 (32%) were actually found to be HBsAg-negative when tested at the HLU laboratory and were instead anti-HBs-positive. Thus, 30 HBsAg-positive patients were finally included in the HLU follow-up.

### 3.3. Specialist Follow-Up

Specialist follow-up was highly successful, as all 30 HBsAg-positive subjects referred concluded the diagnostic, clinical, and therapeutic (where indicated) course. A clinical and laboratory report for the GP was given to each patient at the end of the evaluation and a schedule for the following appointments.

### 3.4. Data of HBsAg Patients Enrolled

Table 2 summarizes clinical, biochemical, and virological data of the 30 patients enrolled. Twenty-seven patients were Italian and 3 were immigrants (2 from Benin and 1 from Brasil). They had a median age of 59.5 years and a median BMI of 27 and were mostly free of significant comorbidities. Among them, a high prevalence of HBV inactive carriers was observed, defined as presence of serum antibodies to HBeAg (anti-HBe), undetectable or low (< 2,000 IU/ml) HBV DNA levels, and normal ALT [11]. HBVDNA was undetectable in 8 patients. No patient showed HCV or HDV coinfection. Liver disease was generally mild, with liver function tests within normal values and no signs of liver disease progression and/or HCC. Liver fibrosis assessment was performed by transient elastography in 15 patients (median 5.4 kPa, range 4.0 - 12). This test was not performed in patients with undetectable HBVDNA or in those who refused. However, all patients started a standard clinical follow-up. Ultrasound evaluation was performed in all patients: a bright liver echo-pattern was observed in 6 patients, a coarse pattern was observed in 2 patients, and signs of portal hypertension (increased portal vein and/or spleen diameter) were present in 3 patients. No other clear alteration in the liver echo-pattern was described in the remaining patients.

Household spread of the virus or surgery and dental procedures were the most common risk factors for HBV infection. More than 30% of the analysed patients consumed alcohol daily. Only 2 patients needed antiviral treatment and were started on entecavir and tenofovir, respectively.

## 4. Discussion

This study shows that, despite our structured attempt to establish a cooperation between GPs and HLU specialists, we failed to effectively identify all HBsAg-positive cared subjects and significantly reduce the number of those not receiving specialist care. In fact, our results highlight the obstacles to an effective primary care detection of hepatitis B infection in an endemic area, the Campania region of Italy.

The major drawback of our bundle was the poor effectiveness of HBV screening. GPs reported that enrolment failure was related to their “excessive workload”, consisting in too many patients to care for with different and complex diseases, coupled with difficulties in practical organization and simultaneous engagement with other ongoing projects in unrelated fields of medicine. After early detecting these difficulties, we extended the number of GPs involved in the program, in the hope that additional GPs would be more successful in their screening efforts. As a matter of fact, only 48 of the 282 anticipated HBV-positive patients were referred overall and only 30 were finally assessed.

Current patients' costs for HBsAg screening in Italy are about 20 euros and have to be paid by those without a formal prior diagnosis of hepatitis B. Only after a formal HBV diagnosis, subsequent costs are covered by the National Health Service. However, considering the number of the GP cared subjects and the metropolitan area involved, it is hardly credible that so few subjects were able to afford these costs. It is instead more likely that patients did not perceive the importance of the health problem we aimed to actively search, possibly due to unfocused information.

When referred, patients were successfully taken on by the HLU. Interestingly, they were mostly found to be HBV inactive carriers. This suggests that most HBV-infected patients who are unaware of their condition have a mild or inactive disease. Notwithstanding, they remain at risk of progressing to a more severe form of liver disease as well as developing hepatocellular carcinoma [12, 13]. In accordance with the latest international guidelines [11], follow-up and possible antiviral treatment should be extended to these patients for whom treatment was previously considered optional. Furthermore, one-third of our patients consumed alcohol on a daily basis. This was probably due to the fact that they were unaware of their liver infection. However, alcohol consumption can definitely increase the risk of liver disease progression and could be prevented by timely diagnosis and management.

In the “scavenged” HBV patients, the most frequent risk factors were related to iatrogenic transmission; this is in agreement with the senior age of most patients and an infection acquired many years before.

Cooperation with GPs is necessary to “scavenge” asymptomatic diseases in the general population. Studies in other settings reported variable results in the screening and evaluation of liver diseases of different etiologies. Some studies identified problems in screening or basic knowledge [7, 8, 14–18], and others obtained very good results [19, 20].

Although our experience was not entirely satisfactory, we are firmly convinced that the involvement of primary care providers in the management of HBV infection is pivotal. Starting from the results of this study, we hypothesize some approaches to implement the outcome of such studies.

(i) Organizing periodic (i.e., every 2-3 months) educational meetings to raise GPs awareness on specific epidemiologic and clinical problems

(ii) Organizing periodic hospital meetings between GPs and specialists to discuss the results obtained and critically assess the ongoing programs

(iii) Alerting the population through media information campaigns, so that they, themselves, ask their GPs for screening and subsequent clinical investigation

(iv) Offering GPs public financial incentives proportional to their enrolment commitment

(v) Offering free screening tests for indigent subjects

(vi) Strengthening the idea of patient well-being and scientific and/or social gratification to stimulate study participation.

GPs could be also selected on the basis of their scientific interest and expertise, making their role more stimulating, as well as their prior workload. Although this could lead to a selection bias, in our current experience, in the absence of a specific interest, the studies could not be feasible and, even if suboptimal, these criteria should be considered when other strategies have failed.

These approaches could be diversely applied in the different geographical settings on the basis of available means and the results obtained could be subsequently evaluated.

Recently, to overcome the difficulties related to the possible involvement of primary care physicians in anti-HCV treatment, it has been proposed to choose subgroups of more expert GPs to help hepatologists and increase the numbers of patients treated [14, 21]. Certainly, treatment calls for a more complex approach than simple screening, and perhaps in some geographical areas we need to work on more basic levels of intervention.

Further studies using new strategies of cooperation are needed to verify the best options for the management of health problems, HBV infection included.

## 5. Conclusions

This program failed in its intent to scavenge chronic HBV-infected patients in the Campania area and to establish a close clinical collaboration between GPs and liver units. GP work pressure, economic difficulties of the patients, and their refusal to participate were the causes most frequently stated by GPs to justify poor enrolment.

The patients referred to the liver units completed their program and most of them were HBV inactive carriers.

New strategies to implement cooperation between GPs and hospital specialists are suggested.

## Figures and Tables

**Table 1:** Declared reasons for low enrolment by the 20 GPs participating in the program.

	**N **(%)
Patients' refusal	2 (10)

High workload	5 (25)

Involvement in other projects	4 (20)

Patient's contribution costs	9 (45)

**Table 2:** Characteristics of 30 patients enrolled.

**General parameters**	

*Median age (range)*	59.5 (32-85)

*Males, n*°* (%)*	15 (50%)

*Median BMI (range)*	27 (22-40)

*Daily alcohol consumers N (%)*	10 (33.3%)

**Biochemical data**	

*Glucose (mean±SD) mg/dl*	93.5±35.6

*AST (mean±SD) IU/dl*	21.75±10.9

*ALT (mean±SD) IU/dl*	24.14±16.1

*GGT (mean±SD) mg/dl*	24.3±13.7

*Bilirubin total(mean±SD) mg/dl*	0.66±0.26

*Bilirubin direct(mean±SD) mg/dl*	0.2±0.07

*Cholesterol(mean±SD) mg/dl*	180.9±53.6

*Triglycerides(mean±SD) mg/dl*	88.13±35.5

*Albumin(mean±SD) mg/dl*	4.3±0.4

*Cholinesterase (mean±SD) mg/dl*	10707±3883

*Creatinine(mean±SD) mg/dl*	0.8±0.24

*Median WBC/mmc (range)*	7.6 (3.0 – 8.7)

*Median PLT/mmc (range)*	203000 (50000- 315000)

**Virological data**	

*HBeAg+ number (%)*	1 (3)

*antiHBe+ number (%)*	29 (97)

*Median HBV DNA (range) IU/ml*	100 (0-1x10^7^)

**Risk Factors for HBV infection**	

*Intravenous drug use, n*°* (%)*	0

*Household contact, n*°* (%)*	7 (23.3%)

*Surgery, n*°* (%)*	23 (76.6%)

*Dental treatment, n*°* (%)*	22 (73.3%)

*Piercing, n*°* (%)*	5 (16.6%)

*Blood transfusion, n*°* (%)*	0

*At risk sexual contact, n*°* (%)*	0

**Hepatocellular Carcinoma, n**°(%)	0

## Data Availability

This manuscript is the description of a cooperative approach between primary care physicians and liver units and offers suggestions to improve this kind of cooperation. Numerical data concern only 30 patients involved in the study and are clearly shown in the manuscript and table.
